# Benefits of Cilostazol’s Effect on Vascular and Neuropathic Complications Caused by Diabetes

**DOI:** 10.3390/medsci13010001

**Published:** 2024-12-24

**Authors:** Diego Castro Musial, Maria Eduarda Ajita, Guilherme Henrique Souza Bomfim

**Affiliations:** 1 Hospital Evangélico de Londrina, Londrina 86015-900, PR, Brazil; 2 Department of Medicine, Pontifícia Universidade Católica do Paraná, Londrina 86067-000, PR, Brazil; mariaeduarda.ajita@gmail.com; 3 Department of Molecular Pathobiology, New York University, New York, NY 10010, USA; ghs5@nyu.edu

**Keywords:** cilostazol, peripheral arterial disease, diabetic neuropathy, diabetes, claudication

## Abstract

Diabetes mellitus (DM) is a global health concern with a rising incidence, particularly in aging populations and those with a genetic predisposition. Over time, DM contributes to various complications, including nephropathy, retinopathy, peripheral arterial disease (PAD), and neuropathy. Among these, diabetic neuropathy and PAD stand out due to their high prevalence and significant impact on patients’ quality of life. Diabetic distal symmetric polyneuropathy, the most common form of diabetic neuropathy, is driven by neuroinflammation stemming from prolonged hyperglycemia. Simultaneously, hyperglycemia significantly increases the risk of PAD, a condition further exacerbated by factors like smoking, age, and sedentary lifestyles. PAD frequently manifests as claudication, a debilitating symptom marked by pain and cramping during physical activity, which limits mobility and worsens patients’ outcomes. Cilostazol, a phosphodiesterase-3 inhibitor, has proven effective in managing intermittent claudication in PAD by improving walking distances and enhancing blood flow. Recent studies have also explored its potential benefits for diabetic neuropathy. Cilostazol’s mechanisms include vasodilation, platelet inhibition, and increased cyclic adenosine monophosphate (cAMP) levels, which may contribute to improved neurological outcomes. However, variability in the clinical evidence due to inconsistent treatment protocols highlights the need for further investigation. This review explores cilostazol’s mechanisms of action and therapeutic applications for managing neuropathy and PAD in diabetic patients, aiming to provide insights into its potential as a dual-purpose pharmacological agent in this high-risk population.

## 1. Introduction

Diabetes mellitus (DM) is a common disease worldwide, with its incidence having progressively increased over the years [1]. The prevalence of diabetes rises as the population ages and is influenced by an individual’s family history [2,3,4]. Over time, patients with diabetes experience numerous complications, including nephropathy, retinopathy, peripheral arterial disease (PAD), and neuropathy, among others [5,6,7,8].

Diabetic neuropathy is a serious complication in diabetic patients due to its high incidence, increased morbidity, and negative impact on quality of life [1,9]. Diabetic distal symmetric polyneuropathy is the most common type of peripheral neuropathy associated with diabetes mellitus. Neuroinflammation, driven by long-term hyperglycemia, contributes to the development of neuropathy [10]. In addition to diabetes-induced neuropathy, hyperglycemia is also a risk factor for developing arterial disease [11]. DM remains a major risk factor for PAD, with diabetic patients having more than double the prevalence of arterial disease compared to that of the general population [11]. This risk is further aggravated when it is combined with other factors such as smoking, age, and a sedentary lifestyle [12]. One of the common clinical symptoms in patients with PAD is claudication, which is characterized in PAD as pain or cramping in the legs during activity due to the reduced blood flow from narrowed arteries, which can limit physical activity and worsen their overall condition [13].

**Figure 1:**
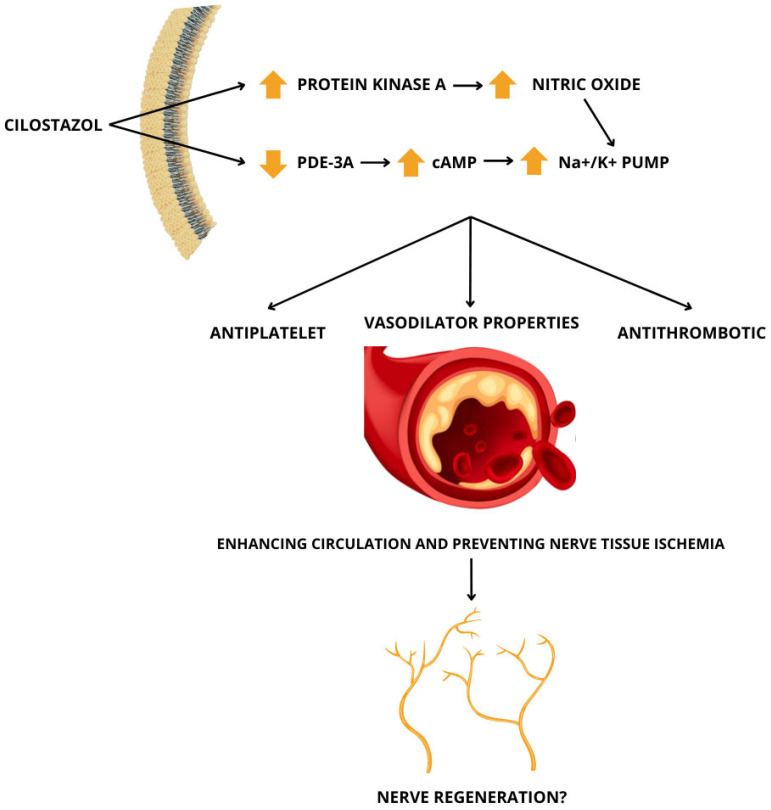
Mechanism of action of cilostazol and its therapeutic potential in the treatment of diabetic neuropathy.

Cilostazol is an effective treatment for the intermittent claudication caused by PAD [14] and has recently been studied for its potential to improve neuropathy [15]. Cilostazol improved both initial claudication distance (pain-free walking distance) and absolute claudication distance (maximum walking distance) compared to that with a placebo [15]. In light of these findings, this review aims to examine the mechanisms and receptors involved in cilostazol’s effects, as they are currently understood, in the treatment of neuropathy and PAD in diabetic patients (Figure 1).

## 2. Diabetic Neuropathy and Cilostazol: What Do We Know?

The development of diabetic neuropathy is hypothesized to involve two primary mechanisms: chronic glucose toxicity and nerve ischemia [16,17]. This condition affects the peripheral nervous system, leading to alterations in sensory, autonomic, and eventually motor axons [18]. Damage to the C and delta B nerve fibers results in reduced sensitivity to temperature and pain. Additionally, patients with diabetic neuropathy may experience balance issues due to the involvement of the A-alpha (α) and A-beta (β) fibers [19]. The progression of neuropathy involves retraction of the axons and reductions in cell bodies and peripheral sensory terminals [18].

The pattern of diabetic neuropathy primarily shows damage to the long sensory axons, progressing in the distal-to-proximal direction. Evidence suggests that in this condition, the damage extends from the cell body to the terminal portion of the axon. However, it remains unclear whether the pathology begins at the axon’s terminal end, involving associated Schwann cells, or originates in the cell body, located in the dorsal root ganglion [18]. Although diabetic neuropathy is not classified as a demyelinating neuropathy, Schwann cells are damaged by chronic hyperglycemia, and severe cases of diabetic neuropathy can exhibit demyelinating features [20].

The mechanisms of neuronal damage in diabetic neuropathy are not fully understood, but evidence suggests that abnormal protein processing, oxidative damage, and mitochondrial dysfunction may contribute to peripheral nerve dysfunction [21,22]. Additionally, alterations in nitric oxide metabolism are believed to lead to perineural vasoconstriction and nerve damage [23]. Excess intracellular glucose increases the polyol pathway, responsible for enzymatic glucose reduction. The heightened activity of aldose reductase leads to elevated levels of sorbitol and fructose, triggering an inflammatory cascade in the metabolic process [24,25]. Moreover, specific changes in the dorsal root ganglion and nerve function include altered spliceosome activity, modifications in the expression of survival motor neuron protein, and upregulation of mRNA processing sites [26].

Among the comorbidities associated with DM, microvascular changes can lead to hypoxia, resulting in the reduced bioavailability of growth factors and nitric oxide. A lack of vascular autoregulation contributes to the development of neuropathy, supporting the hypothesis that nerve ischemia plays a significant role in the pathophysiology of diabetic neuropathy [27].

In 2017, it was estimated that there were 425 million cases of diabetes among adults aged 20 to 79, with a total range of 346 to 545 million. Expanding the age range to include those aged 18 to 99 increased this figure to 451 million cases (8.4%; CI: 7.0–11.2%), with a range of 367 to 585 million. By 2045, these numbers are expected to rise, with projections estimating 629 million people (9.9%; CI: 7.5–12.7%) aged 20 to 79 will be living with diabetes. If this age range is extended to 18 to 99 years, the estimated number rises to 693 million people (9.9%; CI: 7.5–12.9%) who will be affected by the disease [28]. The prevalence of diabetic neuropathy in the general population is reported to be 49% [29], and recent studies indicate a prevalence of approximately 30% [30,31]. The prevalence ranges from 12% in individuals with prediabetes to as high as 90% in patients with diabetes mellitus who are candidates for kidney transplantation [32].

A landmark epidemiological study on diabetic neuropathy was a cohort study conducted in France involving 4400 diabetic patients who were followed from 1947 to 1973. This study found that 50% of the participants developed peripheral neuropathy within 25 years of follow-up [33]. A recent worldwide meta-analysis involving 29 studies and 50,112 participants found that the prevalence of diabetic peripheral neuropathy was greater in individuals with type 2 diabetes than in those with type 1 diabetes [34]. The public health costs associated with diabetic neuropathy include both direct and indirect expenses.

Direct costs cover resources such as hospitalizations, medications, and outpatient care, while indirect costs encompass expenses like transportation, loss of patient income, food, reduced productivity, and financial losses due to disabilities and deaths [35]. Notably, Brazil spends up to USD 52.3 billion on diabetes-related expenses, ranking third among the countries with the highest diabetes-related costs [36]. Globally, the cost of treating diabetes and its complications in patients aged 18 to 99 years old reached approximately USD 850 billion in 2017. For those aged 20 to 79 years old, the estimated cost was USD 727 billion during the same period. By 2024, this figure is expected to increase by 7%, reaching USD 958 billion for patients aged 18 to 99 and USD 776 billion for those aged 20 to 79 [28].

Diabetes and its associated complications account for approximately 12% of global healthcare expenditures [36]. In the United States alone, the annual cost of managing diabetic neuropathy and its related complications is estimated at USD 10 billion [37]. A pilot study by Rosales et al. focused on Filipino patients with type 2 diabetes mellitus and diabetic neuropathy, providing valuable insights into this population’s specific challenges. The participants were divided into three groups: a placebo group (16 patients), a group receiving 100 mg/day of cilostazol (16 patients), and another receiving 200 mg/day of cilostazol (16 patients) [38]. Diabetic neuropathy symptoms were assessed using a neuropathy symptom score (NSS). The diagnosis of neuropathy was confirmed by two neurologists being in agreement, and evaluations of this complication using the NSS occurred at baseline, week 4, week 8, and week 12. To be included in this study, the participants had to have received a diagnosis of type 2 DM more than 3 months prior to the start of the study, an HbA1c level of 8% or less, and continued diabetes treatment throughout the study period. Overall, an improvement in the NSSs was observed when comparing the baseline scores with those at 12 weeks. However, there was no statistically significant difference in the NSSs between the treatment groups at 12 weeks. Therefore, this study did not demonstrate a significant benefit of cilostazol in improving the symptoms of diabetic neuropathy [38].

In another study, 26 patients with type 2 diabetes, stable intermittent claudication, and diabetic peripheral neuropathy were included, consisting of 20 men and 6 women. Diabetic peripheral neuropathy was identified using a vibration perception threshold (VPT) measured with a handheld neurothesiometer on the medial aspect of the first metatarsal head, with the values ranging between 15 and 45 V. The participants were divided into a placebo group (14 patients) and a group receiving 100 mg of cilostazol. Before their inclusion, all participants were treated with antiplatelet and lipid-lowering agents. The effect of the medication on neuropathy was assessed using the Toronto Clinical Neuropathy Scoring System (TCNS) and the VPT over a period of 24 weeks. At the end of this period, there was no statistically significant difference between the placebo group and the cilostazol group based on the TCNS or the VPT measurements for both the right and left feet, indicating that cilostazol showed no benefit in treating diabetic neuropathy [39]. In contrast, the open clinical trial by Okuda assessed the effect of 100 mg of cilostazol on the peripheral arteries in patients with diabetic neuropathy and demonstrated a significant improvement in the blood flow in the dorsalis pedis artery one hour after administering the medication [23]. Based on these findings, the authors suggest that cilostazol may potentially improve diabetic neuropathy by enhancing circulation and preventing nerve tissue ischemia.

The lack of evidence for the benefits of cilostazol in diabetic neuropathy contrasts with initial preclinical and clinical studies [40]. Thus, it is important to note that the literature reports an improvement in diabetic neuropathy among patients treated with a placebo within the first 12 months. Furthermore, the clinical manifestation of worsening neuropathy typically requires observation for more than 1 year [41]. Another potential bias to consider is that despite the development of new neuropathy assessment tools in clinical practice, these tools are not always validated for evaluating the potential benefits of therapeutic interventions [40]. As seen in these studies, the dosage used in clinical trials is either 100 mg/day or 200 mg/day [23,38,39].

Cilostazol is a selective phosphodiesterase-3A (PDE-3A) inhibitor with reversible antiplatelet, antithrombotic, and vasodilatory properties. The inhibition of PDE-3A leads to an increase in cyclic adenosine monophosphate (cAMP) in the intracellular space, which facilitates the drug’s vasoprotective actions [42,43,44,45]. In addition to increasing the cAMP levels in cells, PDE-3A inhibition enhances sodium and potassium (Na^+^/K^+^) pump activity, thereby improving nerve blood circulation, which may be associated with nerve regeneration [46,47,48]. Although the mechanisms by which cilostazol interacts with anti-diabetic and anti-hypertensive drugs have not been fully elucidated, due to its effects, cilostazol may have implications for glucose metabolism and blood pressure regulation [49,50,51]. In diabetes management, cilostazol may enhance the peripheral circulation and insulin sensitivity, potentially augmenting the hypoglycemic effects of drugs like insulin or sulfonylureas, thereby increasing the risk of hypoglycemia [52,53]. Conversely, it may counteract the hyperglycemic side effects of thiazide diuretics or beta-blockers [54,55]. In hypertension, its vasodilatory properties can synergize with anti-hypertensive medications, such as calcium channel blockers or ACE inhibitors, potentially leading to excessive reductions in blood pressure and postural hypotension. Additionally, cilostazol’s reflex tachycardia may complicate therapy with beta-blockers or other rate-modulating drugs [56,57,58]. These interactions underscore the need for careful monitoring and potential dose adjustments when cilostazol is used in patients on these therapies.

Experimental studies using animal models have evidenced an improved motor neuron conduction velocity, along with the restoration of myelinated fiber density and size [46,48,59,60,61]. More specifically, some studies have concluded that cilostazol influences myelin fiber morphology. By blocking neurogenic inflammation and reducing the number of macrophages at the site, this drug appears to promote the supply of cytoskeletal proteins, thereby reducing axon atrophy [48,62]. The effects of cilostazol on the development of experimental diabetic neuropathy were studied in rats with induced diabetes, where this drug enhanced the activity of the Na^+^/K^+^ pump. As a result, alterations in the metabolism of prostaglandins and nitric oxide facilitated improvements in endoneural microvascularization, correcting blood flow and alleviating neural hypoxia [46]. Additionally, the authors reported an increase in the nerve conduction velocity associated with enhanced sodium and potassium pump activity [46]. Yamamoto demonstrated that cilostazol, as an antiplatelet drug with vasodilatory effects, effectively prevented a decrease in the axonal regeneration rate following cryolytic injury to the sciatic nerve in streptozotocin-induced diabetic rats [48]. This study suggested that the improvement in nerve regeneration may be attributed to cilostazol’s ability to mitigate diabetes-related complications such as ischemia and hypoxia [48]. In an analysis of the effects of cilostazol on peripheral neuronal function and structure in diabetic rats, cilostazol inhibited a reduction in the pericyte area in the endoneurial vessels and decreased the expansion of the endoneurial microvessels and the luminal area in relation to the vascular area, both of which are deleterious effects resulting from DM [62].

Based on the assumption that a reduction in cAMP and nitric oxide (NO) is involved in the pathophysiology of diabetic neuropathy, the human neuroblastoma cell line SH-SY5Y was used to investigate the effect of cilostazol on NO production and Na^+^/K^+^ pump activity. Thus, the SH-SY5Y cells were cultured with 5 or 50 mM glucose for 5 to 6 days. They were then exposed to cilostazol or other agents, and their nitrite levels were measured to assess cAMP and Na^+^/K^+^ pump function. In the SH-SY5Y cells cultured in 50 mM glucose, cilostazol significantly increased the NO production and cAMP accumulation in a dose- and time-dependent manner. Additionally, there was a notable recovery of the reduced levels of protein kinase A (PKA) activity in these cells. A PKA inhibitor suppressed the increase in NO elicited by cilostazol, suggesting that this drug facilitates increased nitric oxide production through the activation of PKA [63].

Furthermore, in the cells cultured in 50 mM glucose, the NO agonist L-arginine increased their Na^+^/K^+^ pump activity. By contrast, the NO inhibitor L-NAME inhibited the Na^+^/K^+^ pump in the cells cultured in 5 mM glucose and suppressed the increase in enzyme activity stimulated by cilostazol. These results suggest that NO regulates the activity of the Na^+^/K^+^ pump in the SH-SY5Y cells and that cilostazol enhances this enzyme’s activity by stimulating NO production. Therefore, this study indicates that cilostazol has a beneficial effect on diabetic neuropathy by increasing the activity of the Na^+^/K^+^ pumps, which is directly linked to the elevated production of cAMP and NO in the nerves [63].

## 3. The Effect of Cilostazol on the Circulation of Diabetic Patients

PAD is a common condition in elderly patients, characterized by reduced peripheral circulation. The most common symptom of PAD is intermittent claudication, which manifests as aching, burning, heaviness, or tightness in the leg muscles. These symptoms typically begin after walking a certain distance, walking uphill, or climbing stairs and subside after a few minutes of rest [64]. DM is associated with an increased incidence of PAD, accelerates its progression, and worsens its severity [65]. Several mechanisms contribute to the development of PAD [66], and it is well established that increased free radicals and hypercholesterolemia promote atherogenesis [67]. In particular, the elevated production of reactive oxygen species (ROS) due to oxidative stress, combined with a diminished redox capacity, plays a critical role in the initiation and progression of PAD [68]. In diabetic patients, oxidative stress is heightened by elevated glucose levels, which drive glucose autoxidation, increase glycation, activate advanced glycation end products (AGEs) and their receptors, stimulate the polyol pathway, reduce the glutathione redox cycle, and activate protein kinase C (PKC) [69].

**Figure 2:**
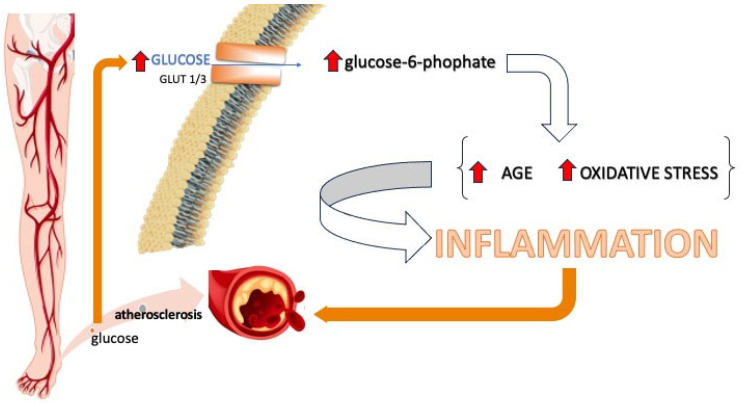
A scheme showing the entry of glucose into the endothelial cells, mediated by the GLUT1 and GLUT3 glucose transporters. Upon entering the cell, excess glucose stimulates an increase in oxidative stress and the activation of advanced glycation end products (AGEs), resulting in an increase in intracellular signaling, stimulating factors that generate inflammation, which contributes to the atherogenesis process.

Glucose enters the vascular endothelium through glucose transporters 1 (GLUT1) and 3 (GLUT3). These transporters facilitate the passive diffusion of glucose along the concentration gradient, meaning they do not rely on insulin for transport [70]. The presence of GLUT1 and GLUT3 explains why hyperglycemia leads to elevated glucose concentrations in the vascular endothelium [71]. An excess glucose concentration in the vascular endothelium leads to an increase in glucose-6-phosphate [72], which activates the nuclear factor kappa B (NF-kB) pathway and stimulates Toll-like receptors (TLRs) [73,74]. These trigger elevated expression of inflammatory cytokines and adhesion molecules, contributing to vascular dysfunction and the development of both microvascular and macrovascular complications in diabetes [75,76]. Moreover, hyperglycemia induces oxidative stress, further amplifying the inflammatory response, a critical factor in the pathogenesis of atherosclerosis in diabetic patients (Figure 2) [24,77].

The entire pathophysiological process of diabetes contributes to the development of PAD, and consequently, the progression of PAD is accelerated in diabetic patients [1,78]. PAD in these individuals can often be asymptomatic due to peripheral neuropathy, delaying a diagnosis until severe complications such as foot ulcers, gangrene, and amputations occur [1,79]. DM is the leading cause of amputations globally, frequently associated with PAD, and diabetic patients have a high likelihood of requiring re-amputation [6,80,81]. The public costs of amputations due to diabetes-related complications impose a significant burden on healthcare systems worldwide. In the United States, expenditures on diabetic foot complications rose from USD 6.6 billion in 2003 to USD 14.8 billion in 2017 [80,82]. This burden is compounded by the costs of post-amputation rehabilitation, including prosthetics and physiotherapy [83].

Given the profound negative impact on the quality of life for diabetic patients and the associated healthcare costs, preventing amputations is crucial [84]. One effective prevention strategy is encouraging walking, which enhances glycemic control and improves cardiovascular health [85]. However, in patients with advanced peripheral arterial disease (PAD), intermittent claudication significantly limits physical activities such as walking [86,87]. Patients often experience intense, transient pain, which hinders their ability to engage in adequate physical activity [88]. Cilostazol has been shown to alleviate this issue, as it effectively improves claudication in PAD patients, including those with diabetes [89,90]. Studies indicate that cilostazol significantly increases the maximum pain-free walking distance for patients suffering from intermittent claudication [80]. Cilostazol has been demonstrated to be more effective than other treatments, such as pentoxifylline, in increasing the maximum walking distance without pain [91]. By inhibiting PDE-3A, cilostazol elevates the cAMP levels in the vascular cells, leading to vasodilation and reduced platelet aggregation, which enhances the blood flow to the lower limbs [92]. Furthermore, cilostazol administration has been associated with the upregulation of vascular endothelial growth factor (VEGF) in the ischemic muscle. Notably, the inhibition of VEGF’s activity significantly diminishes cilostazol-induced angiogenesis [93].

In an experimental study involving rabbits, cilostazol was associated with an 8.0-fold increase in the blood flow in the gastrocnemius during electrical stimulation compared to that in the control group. This effect is attributed to phosphodiesterase inhibition and an increase in adenosine levels [94]. Adenosine, an endogenous nucleoside, plays a crucial role in tissue ischemia physiology [95]; it can inhibit platelet aggregation and enhance tissue perfusion [41,96]. By elevating adenosine levels, cilostazol amplifies these beneficial effects, ultimately improving the walking capacity in patients with PAD [92,95]. Recent studies have demonstrated that the combination of cilostazol with clopidogrel provides advantages in terms of increased walking distance and a reduced risk of adverse events such as stroke and myocardial infarction [90]. Additionally, cilostazol has been shown to improve the amputation-free survival and reduce the long-term mortality in patients undergoing peripheral vascular interventions [97].

## 4. Conclusions

Cilostazol has demonstrated potential as a pharmacological agent for treating both PAD and diabetic neuropathy due to its ability to inhibit phosphodiesterase-3A, promote vasodilation, inhibit platelet aggregation, and increase cAMP levels. While experimental studies suggest that cilostazol may improve the outcomes in diabetic neuropathy, the clinical evidence remains inconsistent, likely due to variations in the dosages, treatment durations, and study designs. The therapeutic benefits of cilostazol for managing claudication in PAD are well established, making it a valuable option in clinical practice. However, the limited availability of effective treatments for diabetic neuropathy, coupled with the high prevalence of PAD in diabetic populations, highlights the urgent need for further research. The current studies on cilostazol’s efficacy in diabetic neuropathy and PAD often include small or heterogeneous populations, limiting the generalizability of their findings. Larger, well-designed trials with standardized methodologies are crucial to address these limitations and provide more conclusive insights into cilostazol’s therapeutic potential. Future research should prioritize clarifying the mechanisms underlying cilostazol’s action, standardizing the treatment protocols, and exploring potential adjunct therapies, such as Vitamin D supplementation, which may help to mitigate the severity of neuropathy. A combined approach could enhance the effectiveness of cilostazol therapy. Additionally, assessing its long-term efficacy in diabetic neuropathy is essential to optimize patient outcomes and establish its role in managing this challenging condition.

## Data Availability

Data are contained within the article.
